# Heart Rate Measurement Using the Built-In Triaxial Accelerometer from a Commercial Digital Writing Device

**DOI:** 10.3390/s24072238

**Published:** 2024-03-31

**Authors:** Julie Payette, Fabrice Vaussenat, Sylvain G. Cloutier

**Affiliations:** Department of Electrical Engineering, École de Technologie Supérieure, Montréal, QC H3C 1K3, Canada; julie.payette.1@ens.etsmtl.ca (J.P.); fabrice.vaussenat@lacime.etsmtl.ca (F.V.)

**Keywords:** heart rate measurement, signal filtering, smart pen, heart rate variability, ECG

## Abstract

Currently, wearable technology is an emerging trend that offers remarkable access to our data through smart devices like smartphones, watches, fitness trackers and textiles. As such, wearable devices can enable health monitoring without disrupting our daily routines. In clinical settings, electrocardiograms (ECGs) and photoplethysmographies (PPGs) are used to monitor heart and respiratory behaviors. In more practical settings, accelerometers can be used to estimate the heart rate when they are attached to the chest. They can also help filter out some noise in ECG signals from movement. In this work, we compare the heart rate data extracted from the built-in accelerometer of a commercial smart pen equipped with sensors (STABILO’s DigiPen) to standard ECG monitor readouts. We demonstrate that it is possible to accurately predict the heart rate from the smart pencil. The data collection is carried out with eight volunteers writing the alphabet continuously for five minutes. The signal is processed with a Butterworth filter to cut off noise. We achieve a mean-squared error (MSE) better than 6.685 × $10^{-3}$ comparing the DigiPen’s computed Δt (time between pulses) with the reference ECG data. The peaks’ timestamps for both signals all maintain a correlation higher than 0.99. All computed heart rates (HR =$\frac{60}{\Delta t}$) from the pen accurately correlate with the reference ECG signals.

## 1. Introduction

Medical devices have become part of our daily lives, especially in the wellness field [1,2]. In the Internet of Things (IoT) era, systems of wireless, interconnected and networked digital devices can now continuously collect, analyze, send and store data over a network, making it tremendously easier to monitor a patient in real time in their living environment [3]. These wearable devices use embedded accuracy sensors to non-invasively monitor healthcare data [4] from the wrist [5], the chest [6], the fingers [7,8] or other parts of the body. The main idea is to perform better medical monitoring during daily activities with precision and safety, all while preserving the patient’s privacy and quality of life. Coupling these wellness devices with emerging data science and machine learning capabilities can provide a more accurate and more comprehensive dashboard of medical indicators [9,10]. This can significantly reduce medical risks by including the patient in the healthcare management loop as early as possible [11]. At the same time, wearable medical devices can simplify the monitoring of patients and keep them at home to improve their quality of life. The improved sensors, micro-controllers and end-point computing framework architectures unleash formidable possibilities for qualitative and predictive patient monitoring [2]. To facilitate the IoT integration in daily lives, a new approach also consists of measuring medical information using smart clothes [12], watches [13], apps and common instruments like pens [14]. The establishment of an easy-to-use surveillance system is an interesting path, especially considering the aging population, the prevalence of cardiovascular disease [15], the emergence of infectious diseases [16] affecting the cardiovascular system and psychological diseases leading to behavioral disorders [17]. Therefore, different medical devices that can measure physiological parameters such as ECG, heartbeat derived from ECG, fetus heart rate [18], respiratory rate (RR), SPO2 [19], temperature [20,21] and blood pressure [22] are vital to monitor fragile patients daily. The choice of sensors is based on the bio-signals to be monitored. Capacitance electrodes are commonly used to detect ECG [23]; respectively, inductance electrodes are used to measure breathing information [24]. Studies show that sets of accelerometers can follow the ribcage movements to accurately predict the respiratory rate as well as the ECG [25]. Some of these sensors can monitor multiple medical parameters simultaneously [26]. For example, the BME680 sensor can record pressure, temperature, humidity and air quality [27], which can be useful in wound monitoring. Most of these wearable multi-parameter medical devices are used to detect heart and lung disorders [1].

The literature already shows that respiratory rate and heartbeat can be inferred from ECG signals [28,29]. Similarly, blood pressure can be measured using PPGs [30] and ECGs [22], allowing for a cuffless blood pressure monitoring that is more comfortable for the patient and can be integrated into a biosensor frame. PPG is a non-invasive optical technique from which many physiological parameters can be derived, as it produces a waveform that correlates with circulatory volume in skin tissue. SPO2 is mainly used to measure PPG using infrared optical sensors usually located on the forehead, finger and more recently on the ribcage [31]. In addition, one of the main applications is to detect dysautonomia and to monitor central nervous system imbalance to assess patients’ behavioral disorders like stress [32]. Dysautonomia [33] describes the imbalance between the sympathetic and parasympathetic nervous system that may be present in anxiety-inducing situations and, more generally, in behavioral disorders. [34,35,36]. It is based on heart rate variability (HRV), which is calculated from the heart rate using the respiratory rate (RR) intervals [37]. This imbalance caused by noxious stimuli can stress homeostasis [38]. Chronic stress causes physical, psychological and behavioral abnormalities by hyperactivating the sympathetic nervous system and deteriorating the patient’s condition. It is accepted that HRV is a good method to monitor and assess the stress status [39]. HRV represents the heart’s ability to respond to a variety of physiological and environmental stimuli [40]. Low HRV is associated with a monotonous, regular heart rate. Furthermore, low HRV has also been associated with impaired regulatory and homeostatic functions of the ANS, which reduces the body’s ability to cope with internal and external stressors. In fact, we can see that accelerometers are often used to detect ECG, heartbeat and respiratory rate, especially located on the wrist to compensate for motion artifacts or the ribcage using respiratory movements [41].

In this study, we demonstrated that the heart rate can be accurately monitored using the built-in accelerometer within a digital smart pen (DigiPen© from STABILO, Heroldsberg, Germany) during the writing process [42,43]. For example, using a pen as a heart rate recording system may present a significant opportunity for monitoring students’ stress in the classroom. This could thereby provide a method of assessing the impact of stress on cognitive abilities [44] and even potentially detect hyperactivity, attention issues or other cognitive and behavioral disorders [45,46]. Dysautonomia also plays a role in detecting cognitive disorders such as Alzheimer’s and Parkinson’s disease [36,47]. As such, a simple act of writing could offer a means to evaluate these cognitive disorders.

## 2. Materials and Methods

### 2.1. Materials

**Figure 1:**
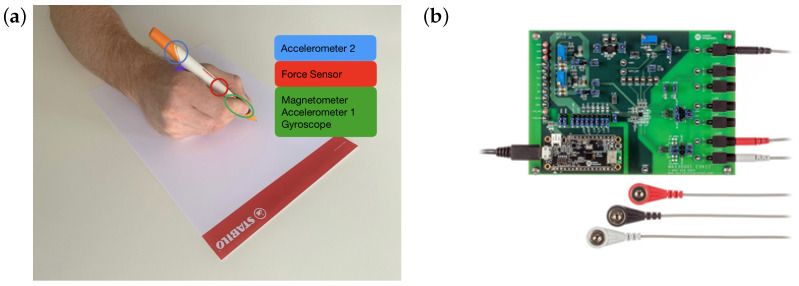
(**a**) STABILO Digipen sensor locations, Copyright by STABILO International GmbH [49]. (**b**) MAX3001 EV kit and electrodes, Copyright by Analog Devices [50]. Used with permission.

**Figure 2:**
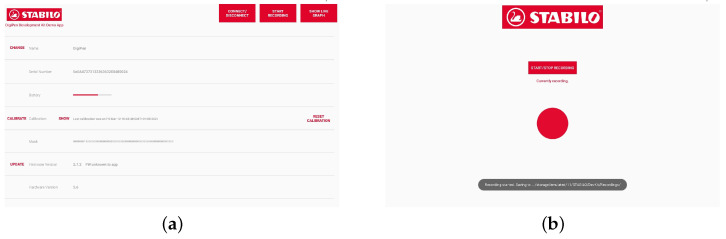
(**a**) DigiPen’s Demo Kit application home page. The ”Connect“ button enables Bluetooth. (**b**) Interface when the data are being recorded from all sensors while writing.

For this study, we worked with the commercially available DigiPen from STABILO [48] (Figure 1a), as well as an ECG evaluation system (Figure 1b). The DigiPen is a smart pen equipped with sensors that is marketed as a tool for assessment of writing motor skills. The pen shown in Figure 1a features two built-in triaxial accelerometers (front and rear), a gyroscope, a magnetometer as well as a force sensor. All data are streamed directly to a connected device with the DigiPen: Development Kit Demo app using a BLE connection. The application’s user interface is shown in Figure 2. Once you connect your pen, you can calibrate it by following the instructions. Afterwards, you only need to select "Record". The data is saved automatically on the device. In order to retrieve the heart rate, we specifically use the accelerometer located near the tip of the pen, where the fingertips rest. It is an LSM6DSL accelerometer with a sampling rate of 100 Hz [49].

**Figure 3:**
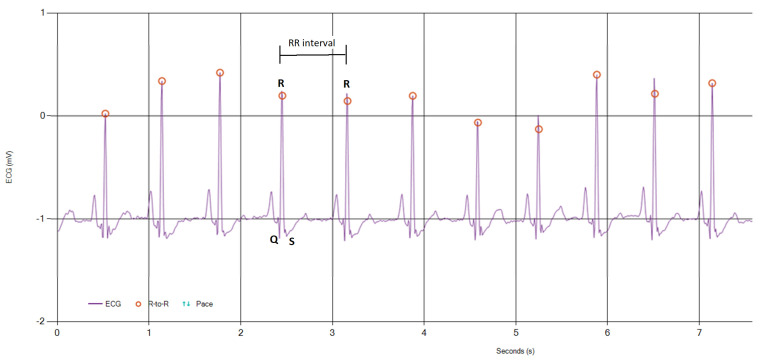
A typical reference ECG signal recorded with the MAX30001 evaluation system. Red dots are the detected R−waves. RR interval is shown as the time period between two R−waves. The QRS complex is the ventricular depolarization.

To evaluate the heart rate predictions from the accelerometer data, we used the MAX30001 [50] evaluation system. It is an assembled circuit that also provides a platform to monitor the data and save them directly in your computer to gather the ECG signal. In our case, we specifically recorded the timestamps of the heart pulses. The time interval between two heartbeats is known as the RR interval, as shown in Figure 3, and is used to measure the heart rate (Equation (1)). There are several components to an ECG signal. They can be separated into segments known as waves [51].

The R-waves correspond to the heart beat. Figure 3 clearly shows the first positive deflection of the QRS complex, which is the ventricular depolarization [52]. Indeed, the R-wave is the highest peak because it is the ventricles’ main mass that depolarizes [51]. Hence, by measuring the time interval between two consecutive R-waves (i.e., the *RR* interval) one can directly compute the heart rate as [53]

(1) $$\mathrm{HR}=\frac{60}{{\Delta t}_{RR}}.$$

However, the *RR* interval can vary between peaks. As such, computing the HR from sequential intervals does not always give the same results. This is because the heart rate can change continuously throughout the day. Those fluctuations between heartbeats are the heart rate variability (HRV) [54].

**Figure 4:**
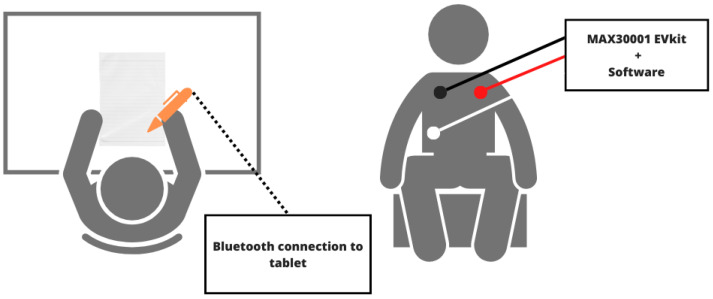
Schematic representation of our experimental configuration: top and front views.

For the data acquisition, we selected eight (8) participants. All participants were in healthy physical condition, while their psychological state was not taken into account or monitored before testing. They wore the MAX30001 EV system’s electrodes on their chest to collect their ECG signal over a five-minute period. In order to minimize the interference, both instruments were connected to their own independent device for data collection. During this time, they were seated in a relaxed position and had to write the alphabet repeatedly on a sheet of paper using the DigiPen. They were asked to hold the pen in the right orientation, with the STABILO logo facing upwards, as shown in Figure 4. Four (4) male participants and four (4) female participants contributed to this preliminary data collection. Only one participant was left-handed. Since the left-handed population is estimated to represent 10–15% of the world population [55], this is deemed a representative sample. This protocol was approved by the institution’s research ethics board.

### 2.2. Methods

To process the signal from the accelerometer, we used Python on Google Colab. First, the anonymous raw data were re-scaled according to the sensor’s specifications provided on STABILO’s website [49]. Then, we combined the 3 separate axes to obtain the Euclidean acceleration trace using the magnitude given as

(2) $$a=\sqrt{{a_{x}}^{2}+{a_{y}}^{2}+{a_{z}}^{2}},$$

where $a_{x},\;a_{y},\;a_{z}$ correspond to the accelerometer’s three components.

**Figure 5:**
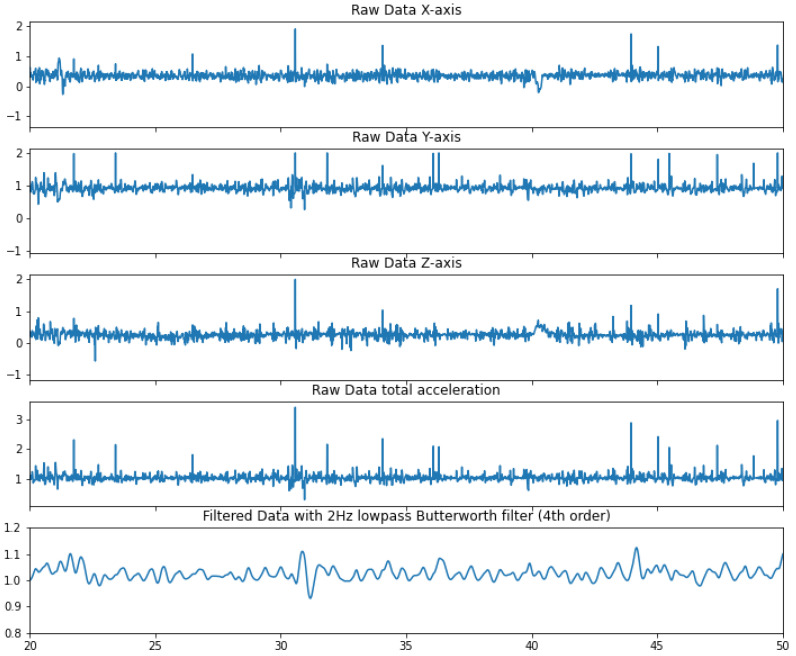
Signal processing of the accelerometer data, plotted on a 30 s period for a more detailed view.

In order to reduce the noise, we applied a fourth-order Butterworth [56] low-pass 2 Hz filter to smooth out the signal, as shown in Figure 5. Finally, the goal was to align the detected peaks from the accelerometer’s signal with the R-waves of the ECG signal. Four vectors were generated from the data. For both the ECG and DigiPen datasets, we created two vectors: (1) a time vector containing the timestamps and the moments of each peak recorded over a five-minute interval (*t*-vector) and (2) a Δt vector containing the time differences between consecutive peaks (Δt-vector). The dimension of these vectors obviously varies with the participants’ heart rate. For a lower heart rate, fewer peaks are detected during a five-minute period than for a higher heartbeat. As seen previously, the heart rate is inversely proportional to the *RR* interval. As such, a larger *RR* interval means a lower heart rate.

## 3. Results and Discussion

**Figure 6:**
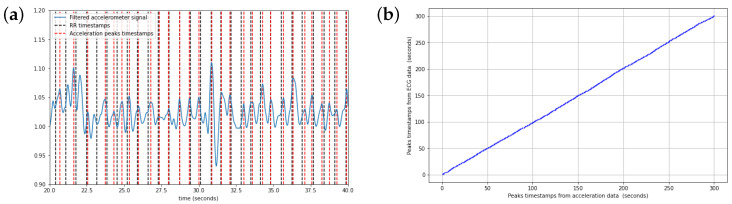
(**a**) DigiPen’s accelerometer signal after filtering with dotted vertical lines indicating the peaks’ timestamps from the accelerometer (-) and the RR interval (-) from the ECG. It is plotted over a 20 s period for a more detailed view. (**b**) Correlation between the peaks’ timestamps.

As seen in Figure 6a, the peaks’ alignment is quite precise. While there is sometimes a delay of less than one second, it does not have a negative impact on our results, since we used the time difference between two pulses to determine the heart rate.

Figure 6 show the typical data obtained for one of our eight (8) participants, but they are highly representative of all our individual test results. Table 1 shows the regularity and consistency of our protocol. Despite the participants having different resting heart rates, we still managed to predict them accurately from the DigiPen’s accelerometer data.

As previously mentionned, for our data analysis, we studied two sets of vectors in the distributions: *t*-vectors and Δt-vectors. As seen in Table 1, the *t*-vectors are practically the same. Both the Pearson correlation coefficients and the cosine similarity factors were computed and suggested over 99% correlation. Meanwhile, the derived average time difference $\bar{\Delta t}$ from the DigiPen data accurately correlates with the reference ECG data. Obviously, since the pen is not as precise as the ECG, the standard deviation for the DigiPen’s results is always larger. However, this does not affect the similarity between the heart rate values. The most considerable deviation recorded is only 0.76 bpm, which is negligible for clinical purposes, since the heart rate is usually expressed to the nearest unit. Welch’s *t*-test was computed between the Δt-vectors to assure that the observed difference between the sample means of the two vectors is statistically significant or if it could have occurred due to random sampling variability. Since all *p*-values are above 0.05, this suggests there is no statistically significant difference. Welch’s t-test was chosen because the distributions’ variances were unequal, but the sample sizes were large (n>250).

**Figure 7:**
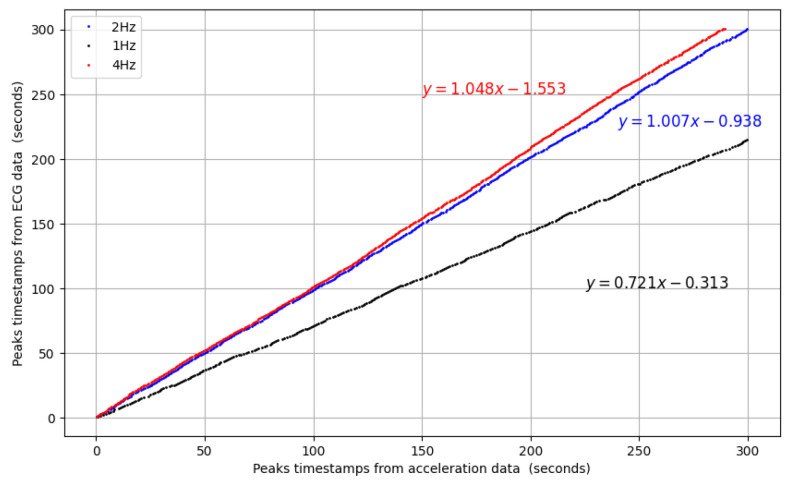
Correlation comparison between the peaks’ timestamps for cut-off frequency low-pass filters 1 Hz(•), 2 Hz(•), 4 Hz(•), with corresponding linear regression equations.

The filtering was optimized through comparison of peaks with the ECG signal. The data collected on the EVkit, which are the RR intervals, give us the number of heartbeats during the test. To find a corresponding number from the accelerometer signal, we programmed a verification loop in Python that tests different cut-off frequencies for the low-pass filter and computes the number of peaks to find the best match. The use of 2 Hz as a cut-off frequency gave the best correlation between both signals for our experiment. Figure 7 shows a comparison of the peaks’ correlation for three frequencies: half our optimal frequency (1 Hz), the optimal frequency (2 Hz) and its double (4 Hz). Although a 1 Hz frequency filter results in a similar correlation, 2 Hz gives a more precise one. For the 4 Hz low-pass filter, we see that the correlation deteriorates rapidly. The linear regression equations for the correlations according to the cutoff frequencies are also shown in Figure 7. The 2 Hz low-pass Butterworth filter provides a linear regression of y=1.004x−0.938, which is the closest to y=x. Also, as mentioned earlier, the heart frequency is not perfectly periodic due to heart rate variability. Therefore, we can use the mean Δt to estimate the samples/period of the pen’s signal. These differ amongst participants, since each cardiac rhythm is unique. From Table 1, $\bar{\Delta t}$ varies between 0.714 s and 1.107 s (equivalent frequencies from 0.9 Hz to 1.4 Hz), which leads to the samples per period varying between 100×0.714=71.4 and 100×1.107=110.7.

**Figure 8:**
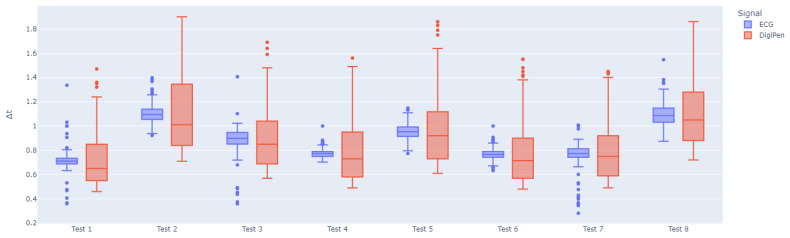
Boxplot comparisons for Δt-vectors from ECG vs. DigiPen on all tests.

We also calculated the mean squared error,

(3) $$MSE=\frac{1}{n}\sum_{j=1}^{n}{\left(Y_{j}-{\hat{Y}}_{j}\right)}^{2},$$

for the eight tests’ Δt-vectors. All are in a $10^{-2}$ range. Finally, Figure 8 shows the comparison boxplots. Although wider spreads can be noticed using the pen’s accelerometer data, the median values are all similar, with a maximal difference of 0.08. The skewness from the pen’s data can be explained by some remaining noise in the signal.

## 4. Conclusions

In clinical settings, the patients’ heart behaviors can and will continue to be monitored with cutting-edge equipment. On the other hand, this work shows the capability of a smart pen’s accelerometer to continuously and precisely determine the heart rate. Indeed, we managed to compute every participant’s heart rate from the pen with a most impressive deviation of only 0.76 bpm. This could prove useful for noninvasive and unbiased behavioral assessments, including heart issues like arrythmia [57], stress [54] or fatigue [58]. Since cardiac rhythms are modulated by the sympathetic and parasympathetic branches of the autonomic nervous system, HRV could also be useful for the early detection and monitoring of Parkinson’s disease evolution in patients [59,60]. Rightly so, future work includes a more detailed dataset and the development of a machine learning algorithm capable of identifying if the participant was under stress or not during the writing process.

## Figures and Tables

**Table 1:** Results obtained from our eight (8) participants. Mean Δt and its standard deviation extracted from both the ECG and Digipen, as well as the mean heart rate. Pearson’s R and cosine similarity factors are computed for *t*-vectors. In contrast, the Welch test’s *p*-value is calculated from the Δt-vectors.

	ECG			DigiPen			Pearson R	Cosine Similarity	MSE (Δt)	Welch’s *t*-Test *p*-Value α=0.05
	$\bar{\Delta t}$ (s)	σ	$\bar{\mathrm{HR}}$ (bpm)	$\bar{\Delta t}$ (s)	σ	$\bar{\mathrm{HR}}$ (bpm)				
test 1	0.715	0.062	83.89	0.714	0.210	83.93	0.999931	0.999979	0.046	0.975
test 2	0.891	0.092	67.33	0.890	0.240	67.38	0.999900	0.999972	0.099	0.960
test 3	1.099	0.072	54.54	1.107	0.308	54.15	0.999886	0.999868	0.068	0.681
test 4	0.774	0.034	77.46	0.775	0.229	77.37	0.999909	0.999975	0.054	0.936
test 5	0.956	0.057	62.75	0.965	0.271	62.14	0.999877	0.999966	0.077	0.551
test 6	0.764	0.043	78.44	0.759	0.224	78.94	0.999870	0.999965	0.053	0.674
test 7	0.769	0.078	77.96	0.777	0.215	77.20	0.999909	0.999976	0.053	0.521
test 8	1.090	0.089	55.03	1.095	0.265	54.76	0.999792	0.999947	0.081	0.754

## Data Availability

The dataset generated and/or analyzed during the current study are available from the corresponding author on reasonable request.

## References

[1] Iqbal S.M.A., Mahgoub I., Du E., Leavitt M.A., Asghar W. (2021). Advances in healthcare wearable devices. NPJ Flex. Electron..

[2] Tsoukas V., Boumpa E., Giannakas G., Kakarountas A. A Review of Machine Learning and TinyML in Healthcare. Proceedings of the 25th Pan-Hellenic Conference on Informatics.

[3] Kelly J.T., Campbell K.L., Gong E., Scuffham P. (2020). The Internet of Things: Impact and Implications for Health Care Delivery. J. Med Internet Res..

[4] Subahi A.F. (2019). Edge-Based IoT Medical Record System: Requirements, Recommendations and Conceptual Design. IEEE Access.

[5] Haescher M., Matthies D.J.C., Trimpop J., Urban B. A study on measuring heart- and respiration-rate via wrist-worn accelerometer-based seismocardiography (SCG) in comparison to commonly applied technologies. Proceedings of the 2nd International Workshop on Sensor-based Activity Recognition and Interaction iWOAR ’15.

[6] Hung P. Estimating respiration rate using an accelerometer sensor. Proceedings of the CSBio ’17: 8th International Conference on Computational Systems-Biology and Bioinformatics.

[7] Alajlan N.N., Ibrahim D.M. (2022). TinyML: Enabling of Inference Deep Learning Models on Ultra-Low-Power IoT Edge Devices for AI Applications. Micromachines.

[8] Pham S., Yeap D., Escalera G., Basu R., Wu X., Kenyon N.J., Hertz-Picciotto I., Ko M.J., Davis C.E. (2020). Wearable Sensor System to Monitor Physical Activity and the Physiological Effects of Heat Exposure. Sensors.

[9] Cruz-Ramos N.A., Alor-Hernández G., Colombo-Mendoza L.O., Sánchez-Cervantes J.L., Rodríguez-Mazahua L., Guarneros-Nolasco L.R. (2022). mHealth Apps for Self-Management of Cardiovascular Diseases: A Scoping Review. Healthcare.

[10] Baldoumas G., Peschos D., Tatsis G., Christofilakis V., Chronopoulos S.K., Kostarakis P., Varotsos P.A., Sarlis N.V., Skordas E.S., Bechlioulis A. (2021). Remote sensing natural time analysis of heartbeat data by means of a portable photoplethysmography device. Int. J. Remote Sens..

[11] Lu L., Zhang J., Xie Y., Gao F., Xu S., Wu X., Ye Z. (2020). Wearable Health Devices in Health Care: Narrative Systematic Review. JMIR MHealth UHealth.

[12] Bryson D., McCann J., Bryson D. (2023). 20—Smart clothing and wearable technology in medical and healthcare applications. Smart Clothes and Wearable Technology.

[13] Yang D., Zhu J., Zhu P. SpO2 and heart rate measurement with wearable watch based on PPG. Proceedings of the 2015 IET International Conference on Biomedical Image and Signal Processing (ICBISP 2015).

[14] Digital Pens Provide New Insight into Cognitive Testing Results. https://neurosciencenews.com/digital-pens-cognitive-test-18868/.

[15] Wimmer N.J., Scirica B.M., Stone P.H. (2013). The Clinical Significance of Continuous ECG (Ambulatory ECG or Holter) Monitoring of the ST-Segment to Evaluate Ischemia: A Review. Prog. Cardiovasc. Dis..

[16] Clerkin K.J., Fried J.A., Raikhelkar J., Sayer G., Griffin J.M., Masoumi A., Jain S.S., Burkhoff D., Kumaraiah D., Rabbani L. (2020). COVID-19 and Cardiovascular Disease. Circulation.

[17] Schneiderman N., Ironson G., Siegel S.D. (2005). Stress and Health: Psychological, Behavioral, and Biological Determinants. Annu. Rev. Clin. Psychol..

[18] Skrivanos A.G., Kosma E.I., Chronopoulos S.K., Kouretas I., Dimopoulos D., Petrakos G., Alhazidou E., Christofilakis V., Ziavra N., Peppas K.P. (2022). Fetus Heart Rate Monitoring: A Preliminary Research Study With Remote Sensing. IEEE Consum. Electron. Mag..

[19] Tamura T. (2019). Current progress of photoplethysmography and SPO2 for health monitoring. Biomed. Eng. Lett..

[20] Boano C.A., Lasagni M., Romer K., Lange T. Accurate Temperature Measurements for Medical Research Using Body Sensor Networks. Proceedings of the 2011 14th IEEE International Symposium on Object/Component/Service-Oriented Real-Time Distributed Computing Workshops.

[21] Basra A., Mukhopadhayay B., Kar S. Temperature sensor based ultra low cost respiration monitoring system. Proceedings of the 2017 9th International Conference on Communication Systems and Networks (COMSNETS).

[22] Chang E., Wang S., Cheng C.K., Gupta A., Hsu P.H., Hsu P.Y., Liu H.L., Moffitt A., Ren A., Tsaur I. Cuff-Less Blood Pressure Monitoring with a 3-Axis Accelerometer. Proceedings of the 41st Annual International Conference of the IEEE Engineering in Medicine and Biology Society (EMBC).

[23] Lim Y.G., Lee J.S., Lee S.M., Lee H.J., Park K.S. (2014). Capacitive Measurement of ECG for Ubiquitous Healthcare. Ann. Biomed. Eng..

[24] Shen C.L., Huang T.H., Hsu P.C., Ko Y.C., Chen F.L., Wang W.C., Kao T., Chan C.T. (2017). Respiratory Rate Estimation by Using ECG, Impedance, and Motion Sensing in Smart Clothing. J. Med. Biol. Eng..

[25] Chan A.M., Ferdosi N., Narasimhan R. Ambulatory respiratory rate detection using ECG and a triaxial accelerometer. Proceedings of the 2013 35th Annual International Conference of the IEEE Engineering in Medicine and Biology Society (EMBC).

[26] Pandian P.S., Mohanavelu K., Safeer K.P., Kotresh T.M., Shakunthala D.T., Gopal P., Padaki V.C. (2008). Smart Vest: Wearable multi-parameter remote physiological monitoring system. Med. Eng. Phys..

[27] Gas Sensor BME680. https://www.bosch-sensortec.com/products/environmental-sensors/gas-sensors/bme680/.

[28] Sarkar S., Bhattacherjee S., Pal S. Extraction of respiration signal from ECG for respiratory rate estimation. Proceedings of the Michael Faraday IET International Summit.

[29] Mirmohamadsadeghi L., Vesin J.M. (2014). Respiratory rate estimation from the ECG using an instantaneous frequency tracking algorithm. Biomed. Signal Process. Control.

[30] Wu W., Wang L., Shen G. (2022). Flexible photoplethysmographic sensing devices for intelligent medical treatment. J. Mater. Chem. C.

[31] Moço A.V., Stuijk S., de Haan G. (2018). New insights into the origin of remote PPG signals in visible light and infrared. Sci. Rep..

[32] Owens A.P. (2016). The Psychophysiology of Dysautonomia. Ph.D. Thesis.

[33] Leti T. (2006). Intérêts de la Variabilité de la Fréquence Cardiaque dans les Dysautonomies. Ph.D. Thesis.

[34] Tulbă D., Cozma L., Popescu B.O., Davidescu E.I. (2020). Dysautonomia in Alzheimer’s Disease. Medicina.

[35] Lo Y.L. (2021). COVID-19, fatigue, and dysautonomia. J. Med Virol..

[36] Goldstein D.S. (2014). Dysautonomia in Parkinson disease. Compr. Physiol..

[37] Shahani B.T., Day T.J., Cros D., Khalil N., Kneebone C.S. (1990). RR interval variation and the sympathetic skin response in the assessment of autonomic function in peripheral neuropathy. Arch. Neurol..

[38] McKendrick T. (1958). Familial dysautonomia. Arch. Dis. Child..

[39] Jovanov E., O’Donnell Lords A., Raskovic D., Cox P., Adhami R., Andrasik F. (2003). Stress monitoring using a distributed wireless intelligent sensor system. IEEE Eng. Med. Biol. Mag..

[40] Rajendra Acharya U., Paul Joseph K., Kannathal N., Lim C.M., Suri J.S. (2006). Heart rate variability: A review. Med Biol. Eng. Comput..

[41] Zhao C., Zeng W., Hu D., Liu H. (2021). Robust Heart Rate Monitoring by a Single Wrist-Worn Accelerometer Based on Signal Decomposition. IEEE Sensors J..

[42] Wehbi M., Hamann T., Barth J., Eskofier B. Digitizing Handwriting with a Sensor Pen: A Writer-Independent Recognizer. Proceedings of the 2020 17th International Conference on Frontiers in Handwriting Recognition (ICFHR).

[43] Oberdorf I.S. (2022). Artificial Intelligence: Digital Pen Helps Persons Learn to Write.

[44] Yaribeygi H., Panahi Y., Sahraei H., Johnston T.P., Sahebkar A. (2017). The impact of stress on body function: A review. EXCLI J..

[45] Hollocks M.J., Howlin P., Papadopoulos A.S., Khondoker M., Simonoff E. (2014). Differences in HPA-axis and heart rate responsiveness to psychosocial stress in children with autism spectrum disorders with and without co-morbid anxiety. Psychoneuroendocrinology.

[46] Denckla M.B. (1996). A theory and model of executive function: A neuropsychological perspective. Attention, Memory, and Executive Function.

[47] Rolinski M., Szewczyk-Krolikowski K., Tomlinson P.R., Nithi K., Talbot K., Ben-Shlomo Y., Hu M.T. (2014). REM sleep behaviour disorder is associated with worse quality of life and other non-motor features in early Parkinson’s disease. J. Neurol. Neurosurg. Psychiatry.

[48] DigiPen Development Kit|STABILO DigiVision. https://stabilodigital.com/digipen-development-kit/.

[49] Sensors|STABILO DigiVision. https://stabilodigital.com/sensors/.

[50] Integrated M., MAX30001 Evaluation System—Evaluates: MAX30001, MAX30002 Technical Report. https://www.analog.com/media/en/technical-documentation/data-sheets/max30001evsys.pdf.

[51] Ashley E.A., Niebauer J. (2004). Conquering the ECG. Cardiology Explained.

[52] Wei X., Yohannan S., Richards J.R. (2023). Physiology, Cardiac Repolarization Dispersion and Reserve.

[53] Prakash E.S., Madanmohan (2005). How to Tell Heart Rate From an ECG? (Learning Objects #769 and #878). Adv. Physiol. Educ..

[54] Zhu J., Ji L., Liu C. (2019). Heart rate variability monitoring for emotion and disorders of emotion. Physiol. Meas..

[55] Sommer I.E.C., Kahn R.S. (2009). (Eds.) Language Lateralization and Psychosis.

[56] Butterworth S. (1930). On the Theory of Filter Amplifiers. Exp. Wirel. Wirel. Eng..

[57] Berntson G.G., Thomas Bigger J., Eckberg D.L., Grossman P., Kaufmann P.G., Malik M., Nagaraja H.N., Porges S.W., Saul J.P., Stone P.H. (1997). Heart rate variability: Origins, methods, and interpretive caveats. Psychophysiology.

[58] da Estrela C., McGrath J., Booij L., Gouin J.P. (2020). Heart Rate Variability, Sleep Quality, and Depression in the Context of Chronic Stress. Ann. Behav. Med. A Publ. Soc. Behav. Med..

[59] Li Y., Wang J., Li X., Jing W., Omorodion I., Liu L. (2021). Association Between Heart Rate Variability and Parkinson’s Disease: A Meta-analysis. Curr. Pharm. Des..

[60] Dorantes-Méndez G., Mendez M.O., Méndez-Magdaleno L.E., Muñoz-Mata B.G., Rodríguez-Leyva I., Mejía-Rodríguez A.R. (2022). Characterization and classification of Parkinson’s disease patients based on symbolic dynamics analysis of heart rate variability. Biomed. Signal Process. Control.

